# Regulatory Role of IL6 in Immune-Related Adverse Events during Checkpoint Inhibitor Treatment in Melanoma

**DOI:** 10.3390/ijms251910600

**Published:** 2024-10-01

**Authors:** Krishna P. Singh, Anuj Singh, Olaf Wolkenhauer, Shailendra Kumar Gupta

**Affiliations:** 1 Department of Systems Biology & Bioinformatics, University of Rostock, 18051 Rostock, Germany; krishna.singh@uni-rostock.de (K.P.S.); olaf.wolkenhauer@uni-rostock.de (O.W.); 2 Amity Institute of Biotechnology, Amity University Uttar Pradesh, Lucknow 226028, India; anuj.singh14@s.amity.edu; 3 Department of Biomedical Engineering & Bioinformatics, Chhattisgarh Swami Vivekananda Technical University, Bhilai 491107, India; 4 Leibniz Institute for Food Systems Biology, Technical University of Munich, 85354 Freising, Germany

**Keywords:** melanoma metastasis, ulcerative colitis, Crohn’s disease, rheumatoid arthritis, integrated bioinformatics analysis, virtual screening, molecular docking, molecular dynamic simulation

## Abstract

The landscape of clinical management for metastatic melanoma (MM) and other solid tumors has been modernized by the advent of immune checkpoint inhibitors (ICI), including programmed cell death-1 (PD-1), programmed cell death-ligand 1 (PD-L1), and cytotoxic T lymphocyte antigen 4 (CTLA-4) inhibitors. While these agents demonstrate efficacy in suppressing tumor growth, they also lead to immune-related adverse events (irAEs), resulting in the exacerbation of autoimmune diseases such as rheumatoid arthritis (RA), ulcerative colitis (UC), and Crohn’s disease (CD). The immune checkpoint inhibitors offer promising advancements in the treatment of melanoma and other cancers, but they also present significant challenges related to irAEs and autoimmune diseases. Ongoing research is crucial to better understand these challenges and develop strategies for mitigating adverse effects while maximizing therapeutic benefits. In this manuscript, we addressed this challenge using network-based approaches by constructing and analyzing the molecular and signaling networks associated with tumor-immune crosstalk. Our analysis revealed that IL6 is the key regulator responsible for irAEs during ICI therapies. Furthermore, we conducted an integrative network and molecular-level analysis, including virtual screening, of drug libraries, such as the Collection of Open Natural Products (COCONUT) and the Zinc15 FDA-approved library, to identify potential IL6 inhibitors. Subsequently, the compound amprenavir was identified as the best molecule that may disrupt essential interactions between IL6 and IL6R, which are responsible for initiating the signaling cascades underlying irAEs in ICI therapies.

## 1. Introduction

**Figure 1:**
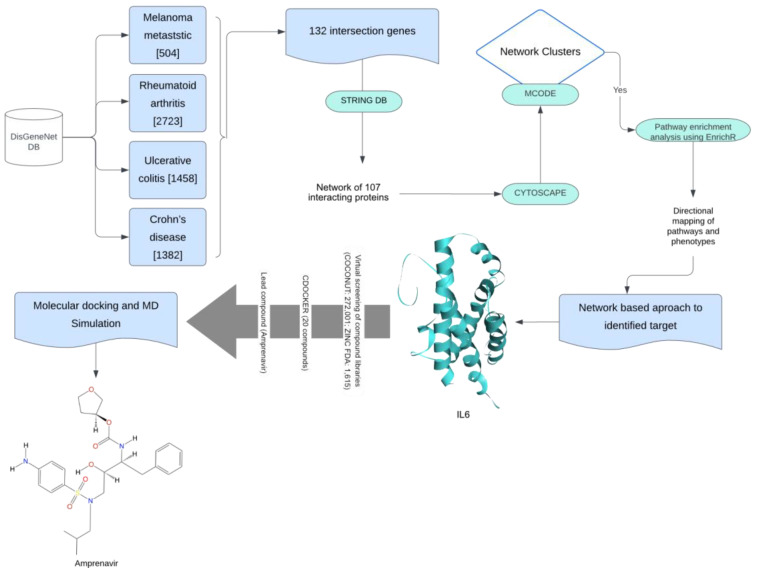
This workflow outlines the process for identifying a lead compound for melanoma and autoimmune disease. The methods utilized were enhanced with various filters. Initially, gene-related information for all diseases was obtained using DisGeNET. The common genes identified were then analyzed through a protein–protein interaction (PPI) molecular map using the STRING database. The resulting PPI network was further analyzed in Cytoscape for cluster identification with MCODE. The most promising cluster underwent enrichment analysis, and we used a network-based approach to identify the target. Virtual screening and molecular docking were employed to find the best compound. Finally, the stability of the lead compound (amprenavir) was assessed through a molecular dynamics (MDs) simulation.

Metastatic melanoma is an advanced and aggressive form of skin cancer that arises from the uncontrolled growth of pigment-producing cells called melanocytes [1]. Immune checkpoint inhibitor (ICI) therapies have emerged as a revolutionary approach in the treatment of melanoma. ICI therapies focus on modulating the immune system to enhance its ability to recognize and attack cancer cells. Various ICIs have been discovered to inhibit specific proteins (e.g., cytotoxic T-lymphocyte-associated antigen 4 (CTLA-4) [2] and programmed cell death protein 1 (PD-1) in tumor-immune environments and thereby eliminate tumor cells [3,4,5,6]. While ICI therapies hold great promise in cancer treatment, they present a distinct challenge by potentially inducing autoimmune phenotypes [7]. The mechanisms that enable the immune system to target cancer cells are also supposed to inadvertently lead to the immune-related adverse events (irAEs) responsible for the exacerbation of autoimmune diseases [8]. This dual nature of immune checkpoint therapies underscores the delicate balance that must be struck between activating the immune system to combat melanoma and preventing it from attacking the body’s tissues, resulting in irAEs [9,10,11,12]. Understanding the crosstalk between melanoma and autoimmune diseases is challenging due to the involvement of a large number of immune cells, and it is vital for the safe use of ICI therapies in the regulation of tumor growth. In this manuscript, we approached this challenge using network-based approaches by constructing and analyzing the molecular and signaling network associated with tumor-immune crosstalk. A detailed workflow of our research is shown in Figure 1.

## 2. Results and Discussion

### 2.1. Protein–Protein Interaction Network at the Interface of Melanoma and Autoimmune Diseases

**Figure 2:**
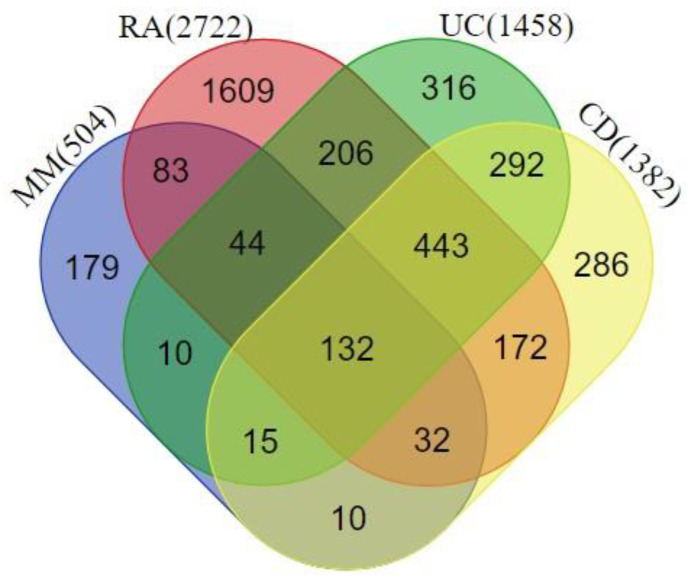
Venn diagram highlighting the overlapping genes between rheumatoid arthritis (RA), ulcerative colitis (UC), Crohn’s disease (CD), and melanoma metastasis (MM). A total of 132 genes were shared among all the disease phenotypes.

We extracted the genes associated with MM (n = 504), RA (n = 2722), UC (n = 1458), and CD (n = 1382) from the DisGeNet database. We found a total of 132 common genes in all 4 disease phenotypes, for which a protein–protein interaction (PPI) network was prepared using the STRING database (Figure 2). We considered these common genes as the connecting links between melanoma and the investigated autoimmune diseases.

**Figure 3:**
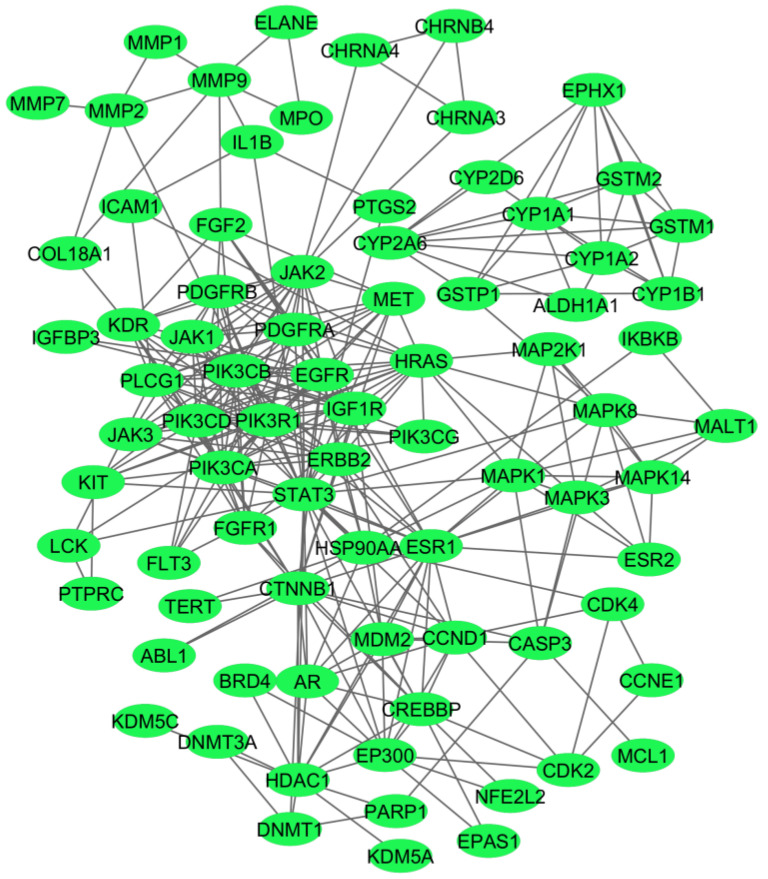
A network of the 107 common genes associated with the investigated four disease phenotypes. The network was prepared using the String database, and the connections between the nodes were above the 0.7 confidence score cutoff.

Out of the 132 genes used for the construction of the PPI network, 25 genes did not exhibit any connections with other genes, and hence, we created a network showing the common 107 genes (Figure 3).

### 2.2. Identification of the Hub Genes from the PPI Network Associated with Crosstalk between Melanoma and Autoimmune Diseases

To identify the key hub genes in the PPI network prepared using the common genes in melanoma and autoimmune diseases, we used the Cytoscape plugin Molecular Complex Detection (MCODE) algorithm. The MCODE algorithm detects interconnected network clusters based on a k-core score that represents the maximal number of connected subgraphs, with all the nodes connected by a minimum number of k degrees [13]. The MCODE algorithm detected six highly connected subnetworks, represented as MCODE modules (Table 1). For the identification of the hub genes responsible for the crosstalk between melanoma and autoimmune diseases, we selected module 1, which contained 16 genes, with the highest MCODE score of 10.13.

### 2.3. Pathway Enrichment Analysis of the Top MCODE Cluster

**Figure 4:**
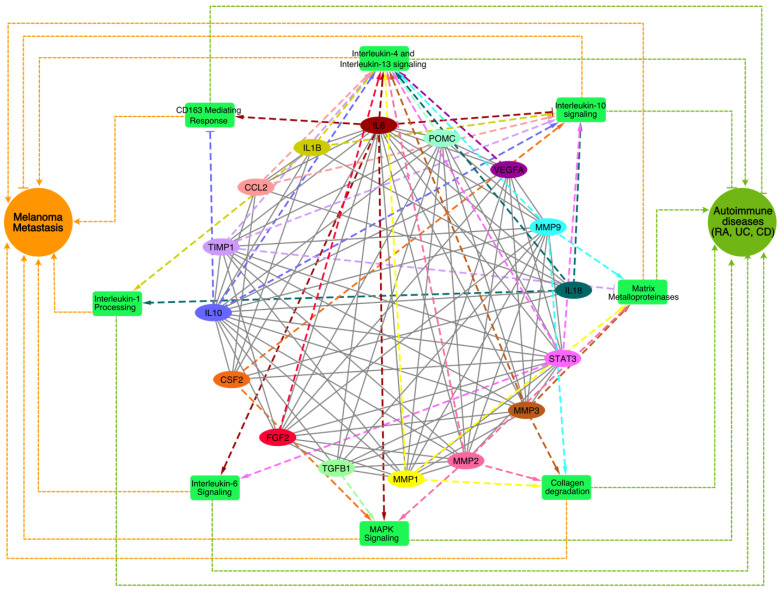
A network of top enriched pathways associated with the genes present in the best cluster was identified through the MCODE analysis. The enriched pathways are shown in the green rectangle boxes, the genes are shown as colored ovals, and the disease phenotypes (MM and autoimmune diseases) are shown as circular nodes. The impacts of the genes on the pathways (dashed lines) and their links to melanoma and the autoimmune disease phenotypes (dotted lines) are shown where the pointed arrowheads indicate ‘activation’ and the blunt-end arrowheads indicate ‘suppression’.

To identify the biological processes and pathways that might play a key role in the crosstalk of melanoma and autoimmune diseases, we performed a pathway enrichment analysis of the genes associated with the top MCODE module using the Reactome database 2022 in the Enrichr web-based server (https://maayanlab.cloud/Enrichr accessed on 10 April 2024). All the enriched pathways (Figure 4), along with the pathway *p*-values, adjusted *p*-values, and gene/protein sets for each case, were examined (Supplementary Table S1).

The interleukin-4 (IL4) and interleukin-13 (IL13) signaling pathways, which play key roles immune regulation and inflammation and are primarily associated with allergic responses and Th2 immunity [14], were among the top enriched pathways. Previous studies have also suggested that these pathways shape the tumor microenvironment and promote tumor progression by modulating immune responses and survival pathways [15]. In autoimmune diseases like RA and UC, IL4 and IL13 contribute to excessive inflammation and tissue damage, fostering autoantibody production and B cell survival [16]. Further, we also found that the interleukin-6 (IL6), interleukin-10 (IL10), and interleukin-1 (IL1) signaling cascades among the top enriched pathways that regulate melanoma [17,18] and autoimmune diseases [19], exerting diverse effects on inflammation, immune dysregulation, and disease progression. Elevated IL6 levels in melanoma correlate with advanced disease stages and therapy resistance, while in autoimmune diseases, IL6 drives inflammation and tissue damage [19]. Conversely, IL10 exhibits dual roles, suppressing anti-tumor immunity in melanoma yet mitigating inflammation in autoimmune diseases. In melanoma, IL1 orchestrates tumor growth, angiogenesis, and metastasis [20] by instigating pro-inflammatory cytokine production, fostering melanoma cell invasiveness, and modulating the tumor microenvironment’s immune cell composition that may contribute to autoimmune disease pathogenesis, fueling inflammation and tissue damage in conditions like RA [21] and IBD [22] by inciting cytokine production and immune cell activation. Additionally, we also found that matrix metalloproteinases (MMPs), collagen degradation, MAPK signaling, and CD163-mediated anti-inflammatory responses were among the top enriched pathways that are known for regulating melanoma and autoimmune diseases [23,24,25,26,27,28,29]. In melanoma, collagen degradation promotes tumor invasion and metastasis by the MMP-mediated breakdown of the extracellular matrix (ECM), facilitating melanoma cell infiltration into surrounding tissues and distant metastasis [30,31]. Elevated levels of collagen degradation products also instigate immune-mediated tissue damage and inflammation in autoimmune diseases like RA [32]. The CD163-mediated anti-inflammatory responses potentially affect the inflammation resolution phase, resulting in progression towards chronic inflammatory phenotypes, together with the exacerbation of autoimmune symptoms. Understanding the intricate interplay of these signaling pathways is crucial for developing targeted therapies that effectively modulate immune responses, together with the management of tumors by immune checkpoint inhibitors.

The pathway enrichment analysis not only helped us to identify the pathways associated with the common genes at the interface of melanoma and autoimmune diseases but also the directions from the genes to the pathways, which enabled us to prioritize the therapeutic targets.

### 2.4. Identification of Lead Molecule and Molecular Docking

IL6 (Figure 4) was found to regulate five pathways among the eight enriched pathways associated with MM and autoimmune disease. This directed network suggests that the inhibition of IL6 will reduce the activity of pathways such as IL6, IL4/IL13 signaling, collagen degradation, CD163-mediating responses, and MAPK signaling, and it may activate IL10 signaling. Thus, the inhibition of IL6 will not only be able to suppress melanoma metastasis but also reduce autoimmune phenotypes, which may be exacerbated during immune checkpoint therapies [33,34,35]. Interestingly, previous reports have also highlighted increased levels of IL6 as a significant contributor to irAEs, as seen in melanoma patients undergoing anti-CTLA-4 ICI therapy [36,37]. Hailemichael and colleagues analyzed samples from ICI-treated tumor patients with immune-related enterocolitis (irEC) and found that IL6 gene expression profiles were more than 24-fold upregulated compared with normal tissues [38]. Lei et al. highlighted that patients with high serum levels of IL6 developed resistance to ICIs [39]. Similarly, average IL6 (both mRNA and protein-level) expression was elevated in RA, UC, and CD patients compared with healthy controls [40,41,42]. These reports have indicated that targeting IL6 will improve the responsiveness of ICIs and also downregulate irAEs.

To identify potential inhibitors for the IL6 protein, we utilized the FDA-approved library using the Zinc15 database (https://zinc15.docking.org/) accessed on 20 April 2024, which contains 1615 compounds, and the natural compound library of the COCONUT database (https://coconut.naturalproducts.net/) accessed on 30 April 2024, which contains 407,270 unique compounds [43]. We filtered the COCONUT database libraries prior to the virtual screening with IL6 for Lipinski’s rule of five [44] in order to consider only drug-like molecules. Only 272,001 compounds were able to pass Lipinski’s rule of five filtering criteria.

The active site of IL6 was selected, considering amino acid residues Phe74, Phe78, Leu178, Arg179, and Arg182, which play a significant role (as hotspot residues) in its interaction with IL6R [45]. We used the LibDock protocol available in the DS2022 for the initial screening of the drug libraries. LibDock is a rigid-based docking program that first calculates hotspots (polar and apolar probes) from the active site of a receptor and then rotates ligands in the cavities for a proper fit [46,47]. The top 20 compounds, based on their LibDock scores [48,49], were further analyzed using the flexible docking tool ‘CDOCKER’ present in the DS2022 (Table 2). In flexible CDOCKER docking [50], both a compound and its receptor can adjust their conformations for a better fit. This adaptability is crucial for accurately predicting the binding affinity and interaction mode between a candidate compound and its target protein [51,52]. Only nine compounds out of twenty could be further docked with IL6 using the CDOCKER protocol.

**Figure 5:**
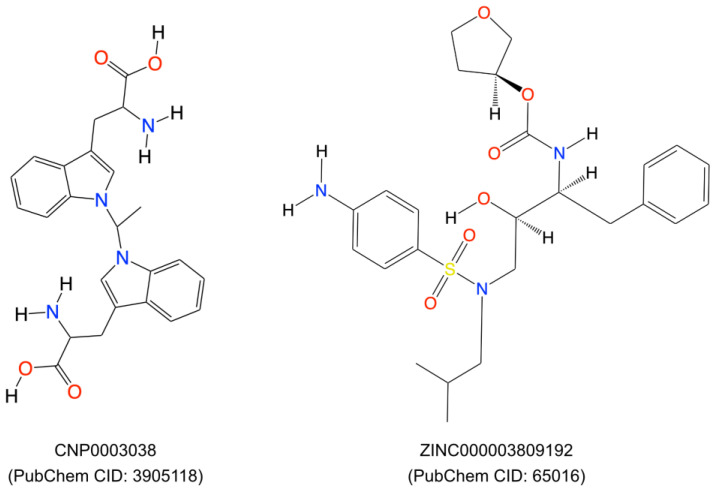
Two-dimensional representation of the top two compounds (ZINC000003809192 and CNP0003038, respectively) which were extracted after virtual screening and molecular docking using the DS20222.

We selected the top two compounds (Figure 5) because they had the highest CDOCKER energy values (CNP0003038: −41.6684 kcal/mol and ZINC03809192: −34.7136 kcal/mol, respectively). One belonged to the natural compound library and the other to the ZINC database.

We performed a literature survey for these two compounds for their toxicity, bioassays, and roles in tumor/autoimmune disease regulation. We found that the compound CNP0003038, also referred to as 1,1′-ethylenebis-L-tryptophan (EBT; PubChem CID: 3905118), enhances the proliferation of EoL-3 eosinophilic leukemia cells and induces the release of eosinophil cationic protein from isolated human peripheral blood eosinophils, resulting in eosinophilia-myalgia syndrome [53]. EBT has also been shown to induce IL-5 production in isolated human T cells, and it induced inflammation, mast cell infiltration, fascia thickening, and adipose tissue fibrosis in an eosinophilia-myalgia-syndrome mouse model [54]. Due to the possible toxic effects of compound CNP0003038 on the immune system, we removed it from further analysis.

The compound from the ZINC database, ‘ZINC000003809192’, also known as amprenavir, is primarily known as a protease inhibitor and used in the treatment of HIV/AIDS. It inhibits the HIV protease enzyme, thereby blocking the cleavage of viral polyproteins into functional proteins, ultimately hindering viral replication [55,56]. Amprenavir was also included in the investigation of FDA-approved small molecule drugs through in-silico screening, and their potential as inhibitors of extracellular signal-regulated kinase (ERK) and apoptosis inducers in MCF-7 human breast cancer cells has been assessed [57]. Based on all the above facts, we selected only Amprenavir for the molecular dynamic simulation.

We employed molecular docking and molecular dynamics simulation analyses to investigate the interaction patterns, stability, and flexibility of the docked complex, which helped us explore the interaction and stability of Amprenavir with IL6 during the simulation. Our molecular docking studies suggested that Amprenavir forms three hydrogen bonds and four hydrophobic bonds with the IL6 amino acid residues SER176, CYS73, MET67, ARG179, LYS54, and LYS66 (more information on the bonds is provided in Supplementary Table S2). To further check if Amprenavir interfered with the binding of IL6 with IL6R, we performed additional protein–protein docking using the HDOCK tool [58]. For this, we first used an IL6 and IL6R complex (PDB ID: 1P9M) [59] and redocked the protein units using HDOCK as a control scenario. Next, we used IL6 in complex with ‘Amprenavir’ and performed the protein–protein docking with IL6R, again using the HDOCK tool. Both scenarios were compared with each other to evaluate the effect of ‘Amprenavir’ on IL6 and IL6R interactions. We observed that the docked complex of IL6-IL6R had a higher docking energy of −398.1 kcal/mol, and thus, it was more stable in comparison to the IL6-Amprenavir-IL6R complex (−262.02 kcal/mol). We further compared the impact of ‘Amprenavir’ on the number of bonds formed between IL6 and IL6R.

The residues essential for IL6 binding to IL6R include the following from IL6: LYS27, GLN28, ARG30, PHE74, PHE75, PHE78, LEU178, ARG179, ALA180, and ARG182 [59], which form various bonds with the following residues from IL6RA: GLU163, GLN190, PHE229, ASP253, GLU277, GLU278, and PHE279, as well as the following residues from IL6RB: LYS118, LYS119, ARG128, VAL167, TYR168, PHE169, VAL230, and VAL264 (more information on the bonds formed between IL6 and IL6R is provided in Supplementary Table S3).

**Figure 6:**
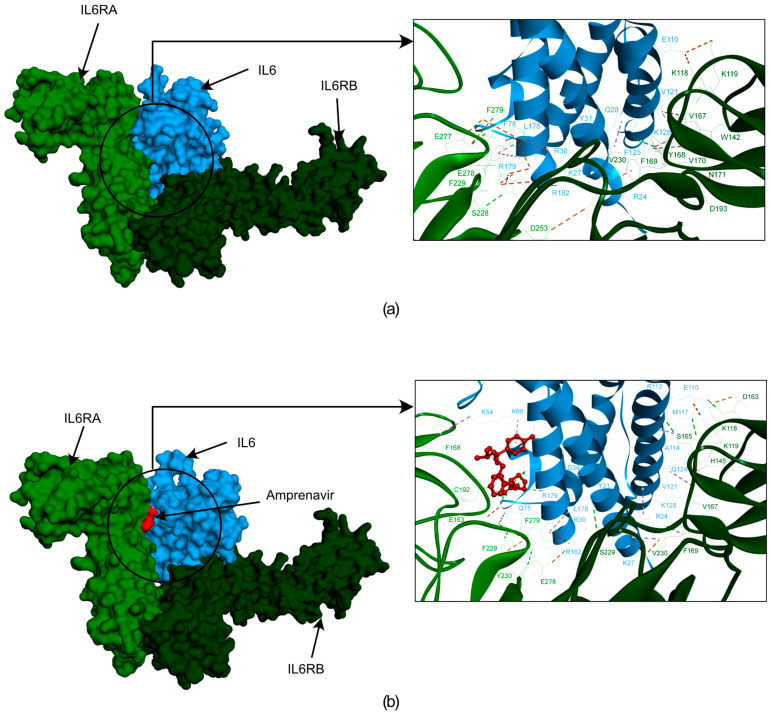
The docked poses obtained from the HDOCK docking tool depicting the interactions between IL6R and IL6 with Amprenavir. In this illustration, IL6R is represented by two different color bases on separate chains (alpha in light green and beta in dark green), while IL6 is shown in blue. The first frame of figure (**a**) showcases the surface representation of IL6R, the IL6 proteins, and their interactions. Figure (**b**) showcases a surface representation of IL6R and the IL6 proteins with Amprenavir. Additionally, the frame provides a zoomed-in version of the surface, highlighting the interactions between IL6R, IL6, and Amprenavir.

Our analysis revealed that the IL6-Amprenavir-IL6R complex lost six significant bonds that were formed between the IL6-IL6R complex. However, one new bond formed between IL6 and IL6R in the presence of Amprenavir (IL6’s ARG179 with IL6RA’s GLU163) (Supplementary Table S3). We also observed that the IL6 amino acid residues LYS66, SER176, and ARG179 and the IL6R residues GLY164 and CYS192 formed bonds with Amprenavir (Figure 6a,b) (Supplementary Table S3). The energies and bond assessments of the docked complex showed that the compound Amprenavir functions as an inhibitor for IL6 interactions with IL6R, disrupting numerous bonds that originally formed between the protein and its receptor.

### 2.5. Molecular Dynamics Simulation

**Figure 7:**
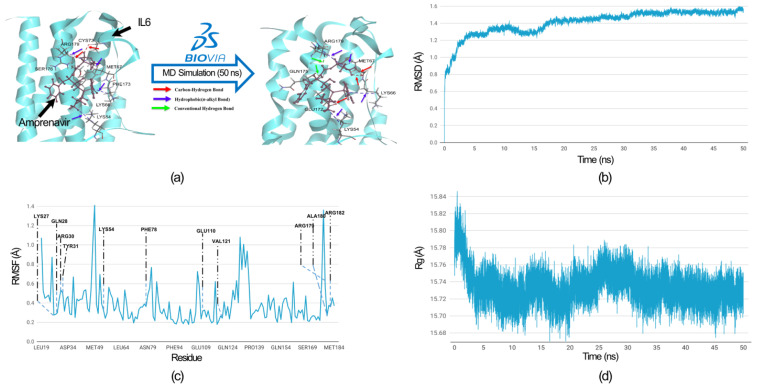
MD simulation analysis of the IL6-Amprenavir complex. (**a**) Hydrogen bonds and hydrophobic interactions between IL6 and Amprenavir are shown before and after the MD simulation. After the MD simulation, Amprenavir formed two additional hydrogen bonds with IL6 compared to the initial docked pose, while the hydrophobic bonds remained unchanged. The colored arrow indicates the nature of the bonds. All IL6 amino acid residues involved in the bond formation are labelled. (**b**) Root Mean Square Deviation (RMSD) graph of the IL6 from the docked complex over a simulation period of 50 nanoseconds (ns). (**c**) Root Mean Square Fluctuation (RMSF) graph of the IL6 interaction site associated with IL6R. The IL6 amino acid residues that directly interacted with IL6R are labelled. (**d**) Radius of gyration (Rg) graph of IL6 from the IL6-Amprenavir complex. The graph suggests that IL6 attained a more compact structure after binding with the drug.

To evaluate the flexibility and overall stability of the IL6-Amprenavir docked complex, we performed time-dependent molecular dynamics (MDs) simulations using the ‘Standard Dynamics Cascade’ protocol in the DS2022. The complex’s stability was assessed through root mean square deviation (RMSD), which measures the deviations of atomic coordinates from their initial positions, and this allowed us to monitor how the structures of the complexes changed over time. In parallel, root mean square fluctuation (RMSF) was used to analyze the flexibility of individual residues, quantifying how much each residue fluctuated during the simulation rather than tracking their positional shifts over time. Additionally, we measured the Radius of gyration (Rg) to assess the compactness of the proteins’ backbones. The Rg measurements provided insights into the overall structural compactness, dynamics, and flexibility of the proteins in a biological environment (Figure 7). We observed the RMSD of the docked complex (IL6-Amprenavir) for 50 ns, and we found that the complex achieved convergence and stabilized at around 20 ns (Figure 7b).

We comprehensively analyzed the residue fluctuations in the RMSF, which were crucial for IL6 to bind with IL6R. The residues within the hydrophilic domain (Lys27, Arg30, Phe78, Arg179, and Arg182) formed salt bridges with the IL6R proteins. In the molecular docking of the IL6-Amprenavir-IL6R complex, we observed the disruptions to the salt bridges involved the residues Lys27 and Arg182 between IL6 and IL6R. The same happened for Phe 78, which disengaged from its bond with IL6R (Supplementary Table S3). All these residues took part in the binding of Amprenavir into the cavities of IL6. During the MD simulation, all these residues showed minimal fluctuations of 0.4 (Å), which indicated that these IL6 residues were tightly engaged with the drug ‘Amprenavir’ and were not available for interaction with IL6R. Furthermore, no residues showed fluctuations of more than 0.7 Å (Figure 7c). In the case of the radius of gyration, during the MD run after the binding of the Amprenavir, IL6 started to achieve a more compact structure (Figure 7d). A comparison of the bonds formed in the IL6-Amprenavir complex before and after the MD simulation is shown in Figure 7a and in Supplementary Tables S2 and S4. The analysis of the final pose of the IL6-Amprenavir complex after the MD simulation indicated increases in the bonds compared to the initial docked pose. Initially, it formed three hydrogen bonds with various IL6 residues, which increased to five after a 50 ns production run. Overall, our analysis indicated that the compound Amprenavir binds with IL6 and forms a stable complex at the same site that is associated with IL6R interactions.

## 3. Methods and Methodology

### 3.1. Data Collection and Protein–Protein Interaction (PPI)

The genes related to MM, RA, UC, and CD were extracted from the DisGeNet database (https://www.disgenet.org/) accessed on 15 March 2024, which is one of the largest publicly available collections of genes and variants associated with human diseases [60]. The data extracted from DisGeNet was subsequently analyzed to identify the common genes among the above-mentioned disease phenotypes. All the common genes were further used to explore their protein–protein interactions using the STRING database (http://string-db.org) accessed on 30 March 2024 [61], with a confidence score cutoff of 0.70.

### 3.2. Identification of Highly Interconnected Clusters in the Tumor-Autoimmune PPI Network

We used the Cytoscape plugin the Molecular Complex Detection (MCODE) algorithm [13] to identify highly connected clusters in the protein–protein interaction network of the common genes associated with the tumors and the selected autoimmune disease phenotypes. The degree cutoff 2 was used to control the genes that were to become part of the cluster. New members were added to the cluster only if their node score deviates from the cluster’s seed node score by less than the set cutoff of 0.2. The k-score cutoff 2 was used to filter out clusters that did not contain a maximally interconnected sub-cluster of a degree score of at least 2. A max depth of 100 was set to limit the distance from the seed node within which MCODE could search for cluster members. This approach allowed us to partition the network based on its topology, pinpointing densely connected regions within the protein–protein interaction network associated with the crosstalk between melanoma and the immune checkpoint therapies that induced irAEs.

### 3.3. Pathway Enrichment Analysis

For the top cluster identified using the MCODE analysis, we identified enriched pathways using Enrichr (https://maayanlab.cloud/Enrichr/) accessed on 10 April 2024 [62]. We specifically focused on the pathways that were present in the Reactome 2022 database, with the significance threshold of a *p*-value of 0.05 for the enrichment analysis. The pathways were sorted on the basics of the combined score [63], which was described as follows:

C=log⁡(p)×z

where *C* is the combined score, *p* is the Fisher exact test *p*-value, and *z* is the z-score for the deviation from the expected rank. Further, we filtered enriched pathways that were previously identified to play a role in melanoma and autoimmune diseases. From the filtered pathways, we identified the nodes that were common in all the enriched pathways for future analysis.

### 3.4. Three-Dimensional Structure Preparation and Screening of the Lead Compounds

We conducted virtual screening and molecular docking analyses to elucidate the inhibition mechanism and identify the potential lead compounds for IL6 inhibition. To perform these analyses, we use a 3D model of IL6 (PDB ID: 1ALU) from the RCSB PDB database (https://www.rcsb.org/) accessed on 15 April 2024 [64]. The model was subjected to the ‘Prepare Protein’ protocols of the Biovia Discovery Studio 2022 (DS2022) [65] using the CHARMM force field [66]. The structure was further optimized using the ‘Smart Minimiser’ algorithm to achieve a stable state through energy minimization. The minimization process was completed in 2000 steps, with an RMS gradient tolerance set at 0.1. With the help of the ‘Receptor-ligand Interaction’ tool of the DS2022, we defined the IL6 binding site based on the amino acid residues that played a key role in its interaction with IL6R [64]. To identify potential inhibitors for the IL6 proteins, we conducted a virtual screening of the natural products using Collection of Open Natural Products (COCONUT), an extensive and well-annotated resource for natural products [43]. Additionally, we included an FDA-approved drug library from the ZINC 15 database [67] for the virtual screening using the ‘LibDock’ protocol of the DS2022. All the docked compounds were further subjected to the flexible ‘CDOCKER’ program in the DS2022 [65,68]. Finally, we performed the protein–protein docking between IL6R and IL6 in the complex with the screened drug molecules using the HDOCK tool [58].

### 3.5. Molecular Dynamic Simulation

We assessed the binding affinity of the IL6 inhibitor docked at the IL6R interaction site, and we performed the MD simulation using the DS2022. The MD simulation was performed in an implicit solvent environment to investigate the stability, conformational changes, and dynamic behavior of the inhibitor in the binding cavity by examining the formation of diverse electrostatic interactions. For the MD simulation, the complex was subjected to an initial minimization phase consisting of 1000 steps using the steepest descent algorithm, followed by an additional 2000 steps employing the conjugate gradient method with the CHARMM force field [66]. After minimization, the systems underwent a heating phase where the initial temperature was incrementally increased from 50 K to 300 K in 50 ps intervals. Subsequently, an equilibration step lasting 100 ps was performed. The adjusted velocity frequency was configured to 50 for the heating and equilibration phases. Subsequently, a production run of 50 ns was conducted within an NVT assembly (maintaining normal volume and temperature) at a constant temperature of 300 K, with the results being saved at intervals of 0.02 ns. For the entire simulation run, we analyzed trajectories consisting of 25,000 conformations. Various properties, including root mean square deviation (RMSD), root mean square fluctuation (RMSF), and the radius of gyration (ROG), were examined using the ‘Analyze Trajectory Protocol’ of the DS2022

## 4. Conclusions

The introduction of ICIs, such as PD-1, PD-L1, and CTLA-4 inhibitors, has revolutionized the clinical management of metastatic melanoma (MM). These inhibitors have shown remarkable efficacy in controlling the growth of tumors. However, their use also leads to irAEs, resulting in the exacerbation of autoimmune diseases such as RA, UC, and CD in melanoma patients. Our research explored the interface between MM and autoimmune diseases, aiming to identify potential druggable targets and understand the crosstalk between these conditions for the safe use of ICIs in MM management. Using an integrative network approach, IL6 was identified as a promising target at the interface between MM and autoimmune diseases. Through structure biology approaches, the lead compounds capable of inhibiting IL6, such as Amprenavir, were identified. As IL6 is mainly an acute inflammatory cytokine, presumably, an IL6 inhibitor may be used to treat the acute inflammatory responses caused by ICIs in melanoma patients that later exacerbate the autoimmune phenotypes. However, further laboratory experiments are needed to validate the efficacy of Amprenavir together with ICIs.

## Figures and Tables

**Table 1:** Network modules generated by MCODE, with their score, number of nodes, and interactions, along with the associated gene names.

Modules	Nodes	Interaction	MCODE Score	Genes
1	16	76	10.133	*CCL2*, *CSF2*, *FGF2*, *IL10*, *IL18*, *IL1B*, *IL6*, *MMP1*, *MMP2*, *MMP3*, *MMP9*, *POMC*, *STAT3*, *TGFB1*, *TIMP1*, and *VEGFA*
2	7	18	6	*CREBBP*, *EP300*, *FOXO3*, *HIF1A*, *MAPK1*, *MDM2*, and *TP53*
3	4	6	4	*AKT1*, *CD40*, *CD40LG*, and *PIK3CG*
4	8	13	3.71	*CTNNB1*, *CXCL10*, *CXCL8*, *IL1A*, *IL4*, *MYC*, *NFKB1*, and *TNF*
5	7	10	3.33	*CALM1*, *CALM2*, *CALM3*, *CXCR4*, *FAS*, *PIK3CB*, and *STAT5A*
6	3	3	3	*HLA-B*, *HLA-C*, and *HLA-DQB1*

**Table 2:** List of top 20 compounds identified after the virtual screening of the IL6 binding site responsible for interacting with IL6R.

S. No.	Compound ID	Database	Compound Name	LibDock Score	-CDOCKER Energy (kcal/mol)
1	CNP0003841	Coconut	N-[(3-methoxyphenyl)methyl]-3-({5-[(4-phenylpiperazin-1-yl)methyl]-1,2-oxazol-3-yl}methyl)oxetan-3-amine	127.506	NA
2	CNP0004058	Coconut	2-chloro-5-hydroxy-N-{[4-hydroxy-5-(hydroxymethyl)-3-{4-[3-(trifluoromethyl)phenyl]piperazin-1-yl}oxolan-2-yl]methyl}benzamide	126.919	NA
3	CNP0004582	Coconut	2-{[({3-methyl-4-[(7-methyl-1H-1,3-benzodiazol-2-yl)methyl]-6-(propan-2-yl)cyclohex-2-en-1-yl}methyl)carbamoyl]methoxy}acetic acid	122.508	18.4652
4	CNP0004629	Coconut	2-{[({3-methyl-4-[(1-methyl-1H-1,3-benzodiazol-2-yl)methyl]-6-(propan-2-yl)cyclohex-2-en-1-yl}methyl)carbamoyl]methoxy}acetic acid	121.359	13.0031
5	CNP0000288	Coconut	7-methoxy-2-(4-methoxyphenyl)-4-[2-(4-methoxyphenyl)ethyl]-3,4-dihydro-2H-1-benzopyran	120.936	25.4486
6	ZINC03809192	ZINC	[(3S)-oxolan-3-yl] N-[(2S,3R)-4-[(4-aminophenyl)sulfonyl-(2-methylpropyl)amino]-3-hydroxy-1-phenylbutan-2-yl]carbamate	120.668	34.7136
7	CNP0004224	Coconut	4-(dimethylamino)-N-[5-hydroxy-7a-(2-{[2-(1H-indol-3-yl)ethyl]carbamoyl}ethyl)-3,3,5-trimethyl-octahydro-1H-inden-1-yl]benzamide	118.314	NA
8	CNP0004392	Coconut	4-[(2-{3-[2-(pyrrolidin-1-yl)pyridin-4-yl]-1,2,4-oxadiazol-5-yl}pyrrolidin-1-yl)methyl]benzoic acid	118.034	NA
9	CNP0003909	Coconut	3-[4-(4-methoxyphenyl)-1H-imidazol-2-yl]-4-[(4-methylphenyl)methyl]morpholine	117.757	17.5344
10	ZINC03955219	ZINC	[(3aS,4R,6aR)-2,3,3a,4,5,6a-hexahydrofuro [2,3-b]furan-4-yl] N-[(2S,3R)-4-[(4-aminophenyl)sulfonyl-(2-methylpropyl)amino]-3-hydroxy-1-phenylbutan-2-yl]carbamate	117.727	18.3056
11	CNP0004686	Coconut	4-cyano-N-{2,3-dihydroxy-5-[6-(morpholin-4-yl)pyridin-3-yl]cyclopentyl}benzamide	117.281	NA
12	CNP0004257	Coconut	N-[(2H-1,3-benzodioxol-5-yl)methyl]-3-({5-[(dimethylamino)methyl]-1,2-oxazol-3-yl}methyl)oxetan-3-amine	116.072	NA
13	CNP0003888	Coconut	3-[4-(4-chlorophenyl)-1H-imidazol-2-yl]-4-[(1-methyl-1H-imidazol-2-yl)methyl]morpholine	115.838	16.6375
14	CNP0004329	Coconut	N-[(2H-1,3-benzodioxol-4-yl)methyl]-3-({5-[(4-phenylpiperazin-1-yl)methyl]-1,2-oxazol-3-yl}methyl)oxetan-3-amine	115.688	NA
15	CNP0004277	Coconut	(5-{[(3-{[5-(pyridin-2-yl)-1,2-oxazol-3-yl]methyl}oxetan-3-yl)amino]methyl}furan-2-yl)methanol	115.539	NA
16	CNP0004720	Coconut	2-{[(3-{[5-(4-methoxyphenyl)-1,2-oxazol-3-yl]methyl}oxetan-3-yl)amino]methyl}phenol	115.352	NA
17	CNP0003796	Coconut	N-[(4-methoxyphenyl)methyl]-3-{[5-(pyridin-2-yl)-1,2-oxazol-3-yl]methyl}oxetan-3-amine	113.167	NA
18	CNP0004058	Coconut	2-chloro-5-hydroxy-N-{[4-hydroxy-5-(hydroxymethyl)-3-{4-[3-(trifluoromethyl)phenyl]piperazin-1-yl}oxolan-2-yl]methyl}benzamide	112.619	NA
19	CNP0003038	Coconut	2-amino-3-(1-{1-[3-(2-amino-2-carboxyethyl)-1H-indol-1-yl]ethyl}-1H-indol-3-yl)propanoic acid	111.568	41.6684
20	CNP0005022	Coconut	4-({3-[4-(pyridin-4-yl)-1H-imidazol-2-yl]morpholin-4-yl}methyl)benzoic acid	111.381	22.286

## Data Availability

The data are contained within the article and the Supplementary Materials.

## Supplementary Materials

The following supporting information can be downloaded at: https://www.mdpi.com/article/10.3390/ijms251910600/s1.
