# Supplementary material for: Regulatory Role of IL6 in Immune-Related Adverse Events during Checkpoint Inhibitor Treatment in Melanoma
Source: Int J Mol Sci. 2024 Oct 1;25(19):10600. doi: 10.3390/ijms251910600 (PMC11476582; doi:10.3390/ijms251910600)
Supplement: Supplementary file 1 [file ijms-25-10600-s001.zip › ijms-3203332-supplementary.pdf]

Supplementary file:

Supplementary Table S1: The table contains 16 genes from the MCODE analysis subjected to pathway enrichment analyses using the Enrichr. In the Enrichr, consider the reactome pathway database for the enrichment. This table contained the name of the pathway and p-value, adjusted p-value and combined score. The last column contained the genes that were enriched in the pathways.

Term	P-value	Adjusted P-value	Combined Score	Genes
Interleukin-4 And Interleukin-13 Signaling	7.72E-31	1.40E-28	103809.1948	*IL10;TGFB1;MMP1;MMP2;MMP3;STAT3; IL18;MMP9;VEGFA;POMC;IL6;IL1B;CCL2;TIMP1,FGF2*
Interleukin-10 Signaling	4.31E-18	1.96E-16	21555.94679	*IL10;IL6;CSF2;IL1B;STAT3; IL18;CCL2;TIMP1*
Matrix Metalloproteinases	3.84E-11	1.16E-09	7769.645942	*MMP1;MMP2;MMP3;TIMP1;MMP9*
Collagen Degradation	2.45E-08	5.58E-07	3236.665389	*MMP1;MMP2;MMP3;MMP9*
CD163 Mediating Response	2.15E-05	2.46E-04	4381.115106	*IL10;IL6*
Interleukin-1 Processing	2.15E-05	2.46E-04	4381.115106	*IL1B; IL18*
Interleukin-6 Signaling	3.29E-05	3.32E-04	3273.111441	*IL6;STAT3*
MAPK Signaling	0.001912227	0.008700632	90.19622528	*IL6;CSF2;MMP2*

Supplementary Table S2: Pre-MD simulation Bond information of the Amprenavir with IL6.

Amprenavir			
**Name**	**Distance**	**Category**	**Types**
IL6:SER176:HA - Amprenavir:O20	2.50039	Hydrogen Bond	Carbon Hydrogen Bond
IL6:CYS73:O - Amprenavir:H63	2.41111	Hydrogen Bond	Carbon Hydrogen Bond
IL6:SER176:O - Amprenavir:H64	2.54729	Hydrogen Bond	Carbon Hydrogen Bond
IL6:MET67 - Amprenavir	5.49327	Hydrophobic	Alkyl
IL6:ARG179 - Amprenavir	5.35202	Hydrophobic	Alkyl
IL6:LYS54 - Amprenavir:C1	5.42493	Hydrophobic	Alkyl
IL6:LYS66 - Amprenavir	4.50905	Hydrophobic	Pi-Alkyl

Supplementary Table S3: The table contained the bond information of the IL6_IL6R complex taken from PBD. The molecular docking was performed with HDOCK to compare IL6_IL6R with IL6_Amprenavir_IL6R. We found that IL6_Amprenavir_IL6R lost 6 bonds compared to the IL6_IL6R.

Interaction of IL6_IL6R complex(PDB)	IL6_IL6R (HDOCK)	IL6_Amprenavir_IL6R (HDOCK)
IL6:ARG30:NH2 - IL6RA:GLU278:OE2	✓	✓
IL6:ARG179:NH2 - IL6RA:GLU277:OE2	✓	✓
IL6RB:LYS118:NZ - IL6:GLU110:OE2	✓	✓
IL6RB:LYS119:NZ - IL6:GLU110:OE2	✓	✓
IL6:LYS27:NZ - IL6RA:ASP253:OD2	✓	✗
IL6:ARG30:NH1 - IL6RA:GLU278:OE1	✓	✗
IL6:ARG179:NH1 - IL6RA:GLU163:OE2	✗	✓
IL6:ARG182:NH2 - IL6RA:GLU278:OE1	✓	✗
IL6:GLN28:NE2 - IL6RB:TYR168:O	✓	✗

IL6:LYS54:NZ - IL6RA:GLN190:OE1	✗	✗
IL6:ARG30:NH2 - IL6RA:PHE279	✓	✓
IL6:PHE78 - IL6RA:PHE229	✓	✗
IL6RB:VAL167 - IL6:VAL121	✓	✓
IL6RB:PHE169 - IL6:LYS27	✓	✓
IL6:TYR31 - IL6RB:VAL230	✓	✓
IL6RA:PHE229 - IL6:ARG179	✗	✗
IL6RA:PHE229 - IL6:ALA180	✓	✗

Supplementary Table S4: Post-MD simulation bond information of the IL6 with Amprenavir.

**Amprenavir (ZINC000003809192)**			
**Name**	**Distance**	**Category**	**Types**
IL6:ARG179:HH11 - : Amprenavir:O19	1.94947	Hydrogen Bond	Conventional Hydrogen Bond
IL6:GLN175:O - Amprenavir:H57	2.1451	Hydrogen Bond	Conventional Hydrogen Bond
IL6:GLU172:OE1 - Amprenavir:H44	2.14798	Hydrogen Bond	Carbon Hydrogen Bond
IL6:MET67:O - Amprenavir:H61	2.74915	Hydrogen Bond	Carbon Hydrogen Bond
IL6:MET67:O - Amprenavir:H62	2.92444	Hydrogen Bond	Carbon Hydrogen Bond
IL6:MET67 - Amprenavir	5.24784	Hydrophobic	Alkyl
IL6:ARG179 - Amprenavir	4.89423	Hydrophobic	Alkyl
IL6:LYS54 - Amprenavir:C3	4.18762	Hydrophobic	Alkyl
IL6:LYS66 - Amprenavir	4.80058	Hydrophobic	Pi-Alkyl
